# Zn(II) can mediate self-association of the extracellular C-terminal domain of CD147

**DOI:** 10.1007/s13238-017-0443-1

**Published:** 2017-08-18

**Authors:** Shujuan Jin, Pengfei Ding, Pengxiang Chu, Hongwei Li, Jianbo Sun, Dehai Liang, Fei Song, Bin Xia

**Affiliations:** 1 Beijing Nuclear Magnetic Resonance Center, Peking University, Beijing, 100871 China; 2 College of Chemistry and Molecular Engineering, Peking University, Beijing, 100871 China; 3 School of Life Sciences, Peking University, Beijing, 100871 China; 4 Cell Engineering Research Center and Department of Cell Biology, State Key Laboratory of Cancer Biology, The Fourth Military Medical University, Xi’an, 710032 China

**Dear Editor,**

CD147 (cluster of differentiation 147), also known as basigin or extracellular matrix metalloproteinase inducer (EMMPRIN), is a cell-surface type I transmembrane glycoprotein expressed at different levels in various cells and tissues, especially at high levels in tumor cells (Grass and Toole, 2015). CD147 plays important roles in multiple physiological processes, such as spermatogenesis, neural network formation, T-cell activation and in the progression of several diseases including tumor metastasis rheumatoid arthritis (RA) atherosclerosis malaria and HIV infection (Muramatsu, 2016).

CD147 was identified to be the cell surface receptor for cytokine cyclophilin A calprotectin S100A9 RH5 of *Plasmodium falciparum* (Muramatsu, 2016). Meanwhile, it was reported that CD147 can function as receptor for itself through self-association (Yoshida et al., 2000; Ding et al., 2017). Subsequent studies revealed that tumor cell-associated CD147 and soluble CD147 can lead to CD147-mediated intracellular signaling (Belton et al., 2008) and stimulate multiple matrix metalloproteinases (MMPs) production in neighbouring cells. MMPs induction was inhibited by specific antibody against multimerized CD147 (Sun and Hemler, 2001). Therefore, self-association is very important for the MMPs-inducing function of CD147.

As a member of the immunoglobulin superfamily (IgSF), CD147 is composed of an extracellular portion (residue 22–205, CD147^EC^) with two Ig domains separated by a 5-residue flexible linker, a single transmembrane domain and a short intracellular domain. There are three conserved N-linked glycosylation sites on CD147^EC^, Asn44 on the N-terminal domain, Asn152 and Asn186 on the C-terminal domain (Grass and Toole, 2015). It is suggested that glycosylated CD147 contained a series of high-mannose and complex-type N-linked glycan structures. Glycosylated CD147 purified from lung carcinoma tissue specimen or produced from eukaryotic expression (residue 32–190) can also stimulate MMPs production (Huang et al., 2013), while prokaryotic expressed CD147 (non-glycosylated) or deglycosylated endogenous CD147 were reported to have no MMP-inducing activity on fibroblasts (Sun and Hemler, 2001; Huang et al., 2013).

The three-dimensional structure of prokaryotic expressed CD147^EC^ was determined by X-ray crystallography (Yu et al., 2008), and it was reported that Lys63 and Ser193 are essential for the dimerization of CD147^EC^ (Cui et al., 2012). However, biochemical studies, including native gel analysis, gel filtration, ultracentrifugation and small-angle X-ray scattering (SAXS), suggest that the prokaryotic recombinant CD147^EC^ exists as a monomer in solution (Schlegel et al., 2009; Chen et al., 2014; Wright et al., 2014).

To clarify the oligomerization properties of CD147, the soluble protein of the extracellular portion of CD147 (CD147^EC^, residue 22–205) was overexpressed and purified from the *E*. *coli* strain origami B (DE3) (Fig. S1A and S1B). The recombinant CD147^EC^ protein was eluted in a single fraction peak as a monomer on size-exclusion chromatography (Fig. 1A, left). We analyzed CD147^EC^ using native PAGE and crosslinking experiments, and only protein band corresponding to monomeric CD147^EC^ could be detected (Fig. 1A, right). Laser light scattering (LLS) showed that the hydrodynamic radius of CD147^EC^ is ~2.3 nm (Fig. 1A, middle), which is in agreement with the size of monomeric CD147^EC^. In addition, no concentration dependent NH signal change in 2D ^1^H-^15^N-HSQC spectra was observed, suggesting no protein self-association. Therefore, the recombinant CD147^EC^ protein only exists as a monomer in solution, consistent with previous studies (Schlegel et al., 2009; Wright et al., 2014).

**Figure 1:**
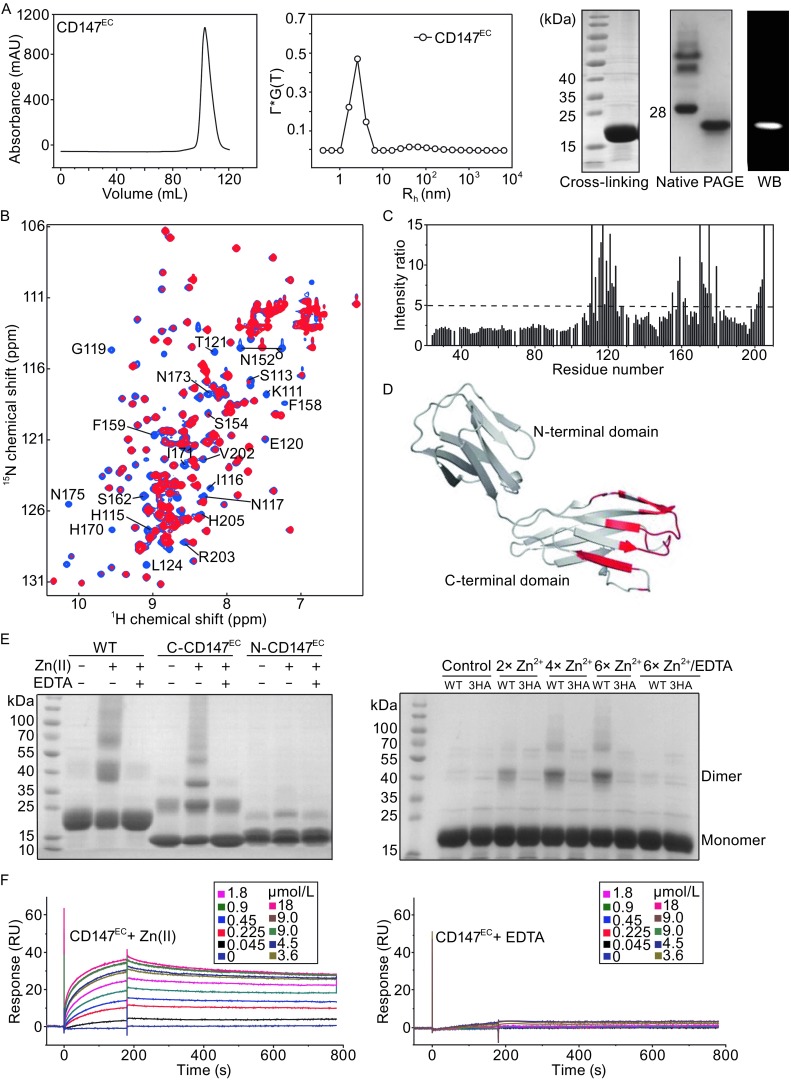
**Zn(II) mediates self-association of CD147** ^**EC**^. (A) Recombinant CD147^EC^ expressed in *E*. *coli* is a monomer in solution. Size-exclusion chromatography of recombined CD147^EC^ (left). Size distribution of CD147^EC^ (middle). Cross-linking, native PAGE, and Western-Blot (WB) results of CD147^EC^ (right). (B) Overlay of 2D ^1^H-^15^N HSQC spectra of CD147^EC^ (0.4 mmol/L ^15^N-labelled protein) with (red) or without (blue) 2-fold excess of Zn(II). Residues displaying significant NH signal intensity decrease upon Zn(II) titration are labeled. (C) Plot of NH signal intensity ratio for CD147^EC^ with and without 2-fold excess of Zn(II). Dashed line represents the ratio of 5. (D) Residues with missing NH signals or the signal intensity reduction ratio larger than 5 upon Zn(II) titration are mapped on the crystal structure of the CD147^EC^ in red (PDB ID: 3B5H). (E) Chemical cross-linking analysis of CD147^EC^ (WT), N-CD147^EC^ and C-CD147^EC^ with or without Zn(II) (left). Chemical cross-linking analysis of CD147^EC^ (WT) and the triple-histidine mutant H115A/H170A/H205A (3HA) with different concentrations of Zn(II) (right). (F) SPR analysis of the self-associations of CD147^EC^ with (left) or without (right) Zn(II). RU represents response unit. A concentration series of CD147^EC^ as analyte (0.045, 0.225, 0.45, 0.9, 1.8, 3.6, 4.5, 9, and 18 μmol/L) with 200 μmol/L Zn(II) or with 1 mmol/L EDTA were injected over the C-CD147^EC^ coated chip for 180 s at a flow rate of 30 μL/min, followed by a 720 s dissociation time

As glycosylation was suggested to play a role in the self-association of CD147, we examined the interaction of CD147^EC^ with different glycans using NMR titration experiments. Three related polysaccharides (N, N’-diacetylchitobiose, 3’-sialyllactose, 3α, 6α-mannopentaose) and five related monosaccharides (sialic acid, D-mannose, D-glucose, glucosamine hydrochloride, D-galactose) were used in NMR titration experiments. Comparison of 2D ^1^H-^15^N HSQC spectra of CD147^EC^, with or without 10-fold excess of glycans, showed that there was no NH signal chemical shift perturbation caused by all the glycans (Fig. S2A–D), except sialic acid (Fig. S3A). Although NH signals of several CD147^EC^ residues were significantly perturbed by sialic acid titration, however, this could be explained by the effect of small pH change due to the addition of sialic acid, as revealed by comparing the 2D ^1^H-^15^N HSQC spectra of CD147 at pH 6.5 and pH 7.0 (Fig. S3B). Taken together, our titration data indicate no direct interaction between the glycans and CD147^EC^.

Unexpectedly, we found that zinc ion could affect NH signals in 2D ^1^H-^15^N HSQC spectra of CD147^EC^ (Fig. 1B). Intensities of some NH signals were significantly weakened with the addition of Zn(II) (Fig. 1B and 1C), which could be reversed by adding EDTA (Fig. S4). Comparing NH signal intensities of free CD147^EC^ and those with 2-fold excess of Zn(II), the N-terminal domain of CD147^EC^ showed an average of ~1.7-fold decrease in NH signal intensity, and the C-terminal domain of CD147^EC^ showed an average of ~4.5-fold decrease. While the changes of NH signal intensity are relatively uniform for residues in the N-terminal domain, some NH signals had over 10-fold intensity decreases for residues in the C-terminal domain. NH signals of residues Lys111, His115-Asn117, Gly119, Thr121, Phe159, His170-Ile171, Asn175, and His205 were even missing, while residues Val110, Ser113, Glu118, Glu120, Ala122-Leu124, Glu155, Ser156, Phe158, Ser161-Ser162, Glu172-Leu174, Met176, Asp179, Arg201-Ser204 had over 5-fold NH signal intensity decrease (Fig. 1C). All these residues are located on one side of the C-terminal domain of CD147^EC^ (Fig. 1D).

As the decrease of NH signal intensities indicating that Zn(II) may mediate self-association of CD147^EC^, we performed chemical cross-linking experiment for CD147^EC^ in the presence of Zn(II). Indeed, strong protein bands corresponding to CD147^EC^ dimer and even high-order oligomers could be detected on the SDS-PAGE (Fig. 1E). The amounts of cross-linked dimer or oligomers were increased with increasing concentration of Zn(II) (Fig. 1E, right). Besides, the cross-linked dimer and oligomers were disappeared when Zn(II) was chelated by EDTA (Fig. 1E). We also performed chemical cross-linking for the two Ig domains alone of CD147^EC^. While small amounts of cross-linked dimers were visible without Zn(II), the amounts of cross-linked dimer and oligomers were significantly increased in the presence of Zn(II) for C-CD147^EC^, with only a slight increase of dimer for N-CD147^EC^ (Fig. 1E, left).

We also carried out surface plasma resonance (SPR) analysis, in which biotinylated C-terminal domain of CD147 ^EC^ was immobilized on SA sensor chips. Sensorgrams with clear association and dissociation processes were observed with the flow though of different concentrations of CD147^EC^ in the presence of Zn(II) (Fig. 1F, left). However, the RU values did not change when EDTA was added into the CD147^EC^ analyte (Fig. 1F, right). Taken together, the chemical cross-linking and SPR data indicate that Zn(II) can mediate self-association of CD147^EC^ mainly through its C-terminal domain, consistent with the NMR titration results mentioned above.

We then determined the solution structure of the C-terminal Ig domain of CD147 (C-CD147^EC^, residues 99–205) using NMR spectroscopy. Detailed restraints and structural statistics of C-CD147^EC^ are summarized in Table S1. The solution structure has been deposited into protein data bank (PDB ID: 5XF0). The solution structure of C-CD147^EC^ comprises nine β-sheets: β1, residues 107–109; β2, residues 113–117; β3, residues 122–128; β4, residues 136–142; β5, residues 147–150; β6, residues 158–163; β7, residues 166–172; β8, residues 181–188; β9, residues 193–203 (Fig. 2A, middle). The solution structure C-CD147^EC^ is essentially the same as the crystal structure (PDB ID: 3B5H), with a RMSD of ~0.7 Å for the backbone heavy atoms in the secondary structure regions (Fig. 2A, right). The relative significant differences between these two structures are mainly in the loop regions linking β-strands (Fig. S6).

**Figure 2:**
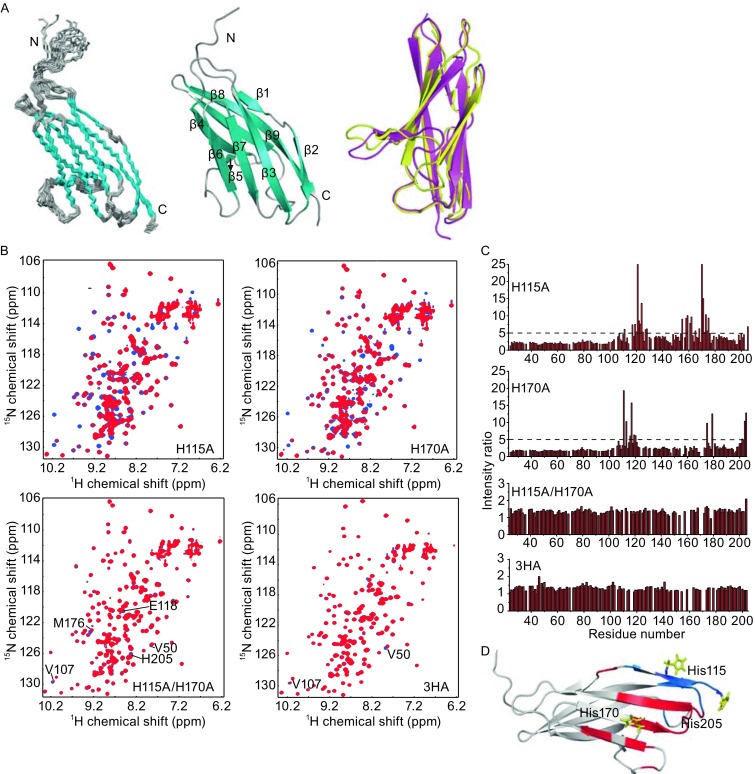
**Solution structure of C-CD147** ^**EC**^ **and interaction between Zn(II) and CD147** ^**EC**^ **mutants**. (A) Solution structure of C-CD147^EC^. Superimposition of the backbone trace of the 20 representative structures of the C-CD147^EC^ (left). The ribbon diagram of the mean structure of C-CD147^EC^ with secondary structural elements labeled (middle). Superimposition of the solution structure (magenta) and the crystal structure (yellow, PDB ID: 3B5H) of the C-CD147^EC^ (right). (B) Overlay of 2D ^1^H-^15^N HSQC spectra of mutants H115A, H170A, H115A/H170A, or H115A/H170A/H205A with (red) or without (blue) 2-fold excess of Zn(II). All samples contain 0.4 mmol/L ^15^N-labelled protein. (C) Plot of NH signal intensity ratio for CD147^EC^ mutants H115A, H170A, H115A/H170A and H115A/H170A/H205A (3HA), between signals with and without 2-fold excess of Zn(II). (D) The NH signal intensity ratio larger than 5 are mapped on the solution structure of C-CD147^EC^. Residues affected by Zn(II) in mutant H115A are colored red, while residues affected by Zn(II) in mutant H170A are colored blue. Sidechains of residues His115, His170, and His205 are shown as sticks and labeled with three-letter amino acid code and residue number

We measured *R*_1_, *R*_2_, and {^1^H}-^15^N NOE values of C-CD147^EC^, and carried out model-free analysis to extract the dynamic parameters for each residue (Fig. S7A). Our results demonstrate that C-CD147^EC^ adopts a rigid structure as reflected by the high {^1^H}-^15^N NOE values (>0.8) for most residues in the secondary structures. The N- and C-terminal regions, loops linking β3 and β4, β4 and β5, and β5 and β6 are relatively more flexible, as reflected by the relatively low {^1^H}-^15^N NOE values (≤0.75).

Model-free analysis shows that residues His102, Lys111, Phe159, Ser161, Gln164, Asn175, and Asp179 display conformational exchanges on the microsecond to millisecond timescales, and some of these residues are just located on the self-association interface of C-CD147^EC^ mediated by Zn(II). Based on *R*_2_/*R*_1_ ratios, the correlation time *τ*_*c*_ for C-CD147^EC^ was calculated to be 7.1 ns, which can be translated into a molecular weight (MW) of ~12 kDa according to previous studies (Rossi et al., 2010). This result further confirms that C-CD147^EC^ (theoretical MW 11.9 kDa) is monomeric in solution. In the presence of Zn(II), *τ*_*c*_ of the C-CD147^EC^ derived from the *R*_2_/*R*_1_ ratio is increased to 9.3 ns (Fig. S7A), corresponding to a molecular weight of over 15 kDa, suggesting that the apparent molecular weight is higher due to Zn(II).

Zn(II) coordination is generally occurring through the side chains of cysteine, aspartate, glutamate or histidine residues of Zn(II)-binding protein. For CD147^EC^, as NH signals of residues His115, His170, and His205 were missing with the addition of Zn(II), we analyzed the tautomeric states of histidines on CD147^EC^ (Fig. S8B). The ^1^H and ^15^N resonances of all histidine sidechains were assigned based on multi-bond ^1^H and ^15^N imidazole signals of 2D ^1^H-^15^N HSQC experiment data, with the help of His to Ala substitutions (Fig. S8D). Based on the positions and intensities of the four multi-bond correlation ^1^H^ɛ1^-^15^N^ɛ2^, ^1^H^ɛ1^-^15^N^δ1^, ^1^H^δ2^-^15^N^ɛ2^, and ^1^H^δ2^-^15^N^δ1^ signals (Pelton et al., 1993), we found that imidazole rings of His102, His115, and His170 adopt the N^ɛ2^-H tautomer, while those of His53 and His205 are in charged tautomeric state (Fig. S8B). We further monitored the signals of the histidine side-chain in 2D ^1^H-^15^N HSQC when CD147^EC^ was titrated by Zn(II). It is obvious that signals of His115, His170, and His205 are also missing due to the addition of Zn(II), while His53 and His102 are not affected by Zn(II) (Fig. S8C). As His205 sidechain exists in charged state which can not provide coordination for Zn(II), His115 and His170 in the C-terminal domain of CD147^EC^ are likely to be candidates for the coordination of Zn(II).

We then generated a series of CD147^EC^ mutants. NMR titration experiment data revealed that H115A mutation only decreased the NH signal responses to Zn(II) for residues around His115 and His205 (Fig. 2B–D), while H170A mutation only decreased the NH signal responses to Zn(II) for residues around His170 (Fig. 2B–D). For H115A/H170A double mutant, we no longer observed the significant NH signal responses towards Zn(II), with only a few NH signals showed small chemical shift perturbation, such as E118, M176, and H205 (Fig. 2B and 2C). Then we generated a mutant with H115A/H170A/H205A triple-histidine mutations, which almost completely abolishes the response of NH signals to Zn(II) in 2D ^1^H-^15^N HSQC spectra, except that a few peaks (such as Val50 and V107) show minor shift (Fig. 2B and 2C). It seems that His205 also participates in Zn(II) interaction, although it should play a minor role. Consistently, chemical cross-linking of H115A/H170A/H205A mutant did not show dimer or oligomer bands on the SDS-PAGE in the presence of Zn(II) at different concentrations (Fig. 1E, right). Therefore, mutating all three histidines into alanines completely abolishes Zn(II) mediated self-association of CD147^EC^, and His115 and His170 are critical residues for the interaction with Zn(II) and self-association of CD147^EC^. As the three histidines are all located on the C-terminal domain, it indicates that only the C-terminal domain is responsible for the self-association of CD147^EC^, while the N-terminal domain is not involved.

A number of studies have indicated that the self-association of CD147 is indispensable for its biological functions, and it is also suggested that glycosylation plays important roles in the self-association of CD147 (Grass and Toole, 2015). However, the molecular mechanism for the self-association of CD147 remains unclear, while biochemical studies resulted in contradictory results on the oligomerization of unglycosylated recombinant CD147 extracellular portion (Yu et al., 2008; Schlegel et al., 2009; Wright et al., 2014). In this study, we have provided chemical cross-linking, size-exclusion, native PAGE, and SPR evidences to demonstrate that unglycosylated CD147^EC^ is incapable of self-association and exists only as a monomer in solution, confirming the previous report (Schlegel et al., 2009). Meanwhile, we also showed that it is unlikely that glycans can mediate the self-association by directly interacting with CD147.

Most importantly, we report for the first time that Zn(II) can directly interact with the extracellular C-terminal domain of CD147 and mediate its self-association. While the solution structure of the extracellular C-terminal domain alone of CD147 is roughly the same as the crystal structure. We identified that His115 and His170 are critical to the Zn(II) interaction and self-association, and very likely participate in Zn(II) coordination. As the two histidines are far away from each other, and our mutagenesis study results suggest that their interactions with Zn(II) are independent of each other, there probably exists more than one Zn(II) mediated dimer interface on the extracellular C-terminal domain of CD147.

As an essential trace metal for living organisms, zinc is involved in many cellular processes, and the recent studies have revealed that zinc ion plays important signaling roles both intracellularly and extracellularly (Hirano et al., 2008). More investigations are needed to reveal the biological roles of Zn(II) induced CD147 self-association, and its molecular mechanism in the function of CD147.

## Electronic supplementary material

Below is the link to the electronic supplementary material.

Supplementary material 1 (PDF 1830 kb)
