# Supplementary material for: Zn(II) can mediate self-association of the extracellular C-terminal domain of CD147
Source: Protein Cell. 2017 Aug 18;9(3):310–5. doi: 10.1007/s13238-017-0443-1 (PMC5829271; doi:10.1007/s13238-017-0443-1)
Supplement: Supplementary file 1 — Supplementary material 1 (PDF 1830 kb) [file 13238_2017_443_MOESM1_ESM.pdf]

MATERIALS AND METHODS

Construction of Plasmids

The cDNA fragments encoding CD147^{EC}, N-terminal Ig domain (N-CD147^{EC}, residue 22-102) and C-terminal Ig domain (C-CD147^{EC}, residue 99-205) were inserted into pET21a (Novagen) with *NdeI* and *XhoI* by standard procedures. These constructs were transformed into origami B (DE3) chemically competent cell, producing soluble proteins. The expressed proteins don't include a hexahistindine tag.

Protein expression and purification

The bacteria were grown overnight in 40 mL LB medium containing 100 μg/mL of ampicillin sodium, 50 μg/mL of kanamycin sulfate and 5 μg/mL of tetracycline at 35°C and then were transferred into 1 L LB medium for growth until the OD_{600} of the medium reached 1.0. Cells were harvested and resuspended in 250 mL M9 medium with 4 g/L $^{13}C_6$-glucose and 1 g/L $^{15}NH_4Cl$ for ^{15}N and ^{13}C isotopic labeling. Incubated cells for another 30 minutes at 35 ℃, then added isopropyl-b-D-thiogalactoside (IPTG) at final concentration of 0.5 mM and incubated cells for another 20 hours at 18℃. The cells were harvested, resuspended in lysis buffer. The lysis buffer of CD147^{EC} is 50 mM Tris-HCl, 50 mM NaCl, pH 8.5 and that of C-CD147^{EC} domain is 50 mM Tris-HCl, 15 mM NaCl, pH 9.5. The cells were lysed by freezing and thawing, followed by sonication. The supernatant of cell lysate was applied onto the ion exchange chromatography column which was derivatized with DE-52. Protein was eluted using linear gradient elution methods and further purified by gel filtration with a Superdex 75 column (Amersham), using 20 mM MOPS, 50

mM NaCl (pH 7.0). $CD147^{EC}$ and $C\text{-}CD147^{EC}$ domain were concentrated and quantified for further experiments.

Chemical cross-linking experiments

Protein (75 μM) with or without Zn(II) (150 μM, 300 μM or 450 μM) was cross-linked with 150 μM ethylene glycolbis (succinimidyl succinate) (EGS) (Pierce) in the reaction buffer (20 mM MOPS, 50 mM NaCl, pH 7.0). Before cross-linking by EGS, EDTA was added with the final concentration of 8 mM when it was used. The reaction mixture was incubated at room temperature for 30 min, and then the reaction was quenched by adding Tris-HCl (1 M, pH 7.5) to a final concentration of 100 mM for 30 min.

Laser light scattering (LLS)

Samples contained 50 μM $CD147^{EC}$ in 20 mM MOPS buffer (pH 7.0), along with 50 mM NaCl and were filtered (0.22 μm) to remove dust prior to measurements. Dynamic light scattering (DLS) was conducted using A commercialized spectrometer equipped with a BI-200SM Goniometer and a BI-Turbo-Corr Digital Correlator. A solid-state laser (100 mW, 532 nm, Changchun, China) polarized at the vertical direction was used as the light source.

NMR titration experiments of Zn(II)

The NMR samples all contained 0.4 mM uniformly ^{15}N labeled protein in 20 mM MOPS, 50 mM NaCl (pH 7.0) with 90% H_2O/10% D_2O. A series of 2D ^{1}H-^{15}N HSQC spectra with gradually increased Zn(II) concentration (0.2 mM, 0.4 mM, 0.8 mM) were collected at 298 K on a Bruker Avance 700 MHz spectrometer (Bruker,

Germany) with cryoprobes. An excess of EDTA (4 mM) was added to the NMR sample for the final 2D ^{1}H-^{15}N HSQC spectrum.

Surface plasmon resonance (SPR) assay

Surface plasmon resonance assays were performed using the Biacore T200 with streptavidin (SA) sensor chip. The biotinylated C-terminal domain (C-CD147^{EC}) was immobilized on a SA sensor chip via biotin-streptavidin interaction. A concentration series of CD147^{EC} as analyte (0.045 μM, 0.225μM, 0.45 μM, 0.9 μM, 1.8 μM, 3.6 μM, 4.5 μM, 9 μM and 18 μM) with or without 200 μM Zn(II) was injected over the C-CD147^{EC}-coated chip for 180 s at rate of 30 μL/min, followed by a 720 s dissociation time. The sample without Zn(II) was also add 1 mM EDTA for SPR experiments. The chip surface was then regenerated with a pulse of glycine-HCl (pH 2.0) at the end of each cycle. All experiments were performed at 25°C in 10 mM HEPES (pH 7.4), 150 mM NaCl, 0.05% P_{20}.

Analysis of histidine tautomer and protonation state

Before NMR data collection on samples in H_2O, 0.4 mM CD147^{EC} was dissolved in 400 μL of a 90% H_2O /10% D_2O solution containing 50 mM NaCl and 20 mM MOPS ((pH 7.0). To study temperature dependence of the NMR signals of CD147^{EC}, 2D ^{1}H-^{15}N HSQC spectra of CD147^{EC} were acquired at 288K, 298K and 308K. To study the effect of Zn(II) on CD147^{EC}, 0.4 mM Zn(II) was added into the NMR sample. NMR data were acquired on Bruker Avance 700 and 800 MHz NMR (Bruker, Germany) spectrometers with cryoprobes. For 2D ^{1}H-^{15}N HSQC experiments, the delay during which ^{15}N and ^{1}H signals become antiphase was set to 22 ms to

refocus magnetization arising from J_{NH} coupling. The ^{1}H transmitter was set to 4.77 ppm and the ^{15}N carrier was set to 205 ppm.

The side-chain of histidines should adopt one of the three tautomeric states ($N^{\varepsilon2}$-H, $N^{\delta1}$-H, and charged), which can be distinguished based on cross-peak patterns and relative peak intensity in 2D ^{1}H-^{15}N HSQC spectrum of the imidazole group (Fig. S8A). For His205, the chemical shifts of $^{15}N^{\delta1}$ (193.1 ppm) and $^{15}N^{\varepsilon2}$ (177.62 ppm) are very close, and all 4 $^{1}H^{\varepsilon1}$-$^{15}N^{\varepsilon2}$,$^{1}H^{\varepsilon1}$-$^{15}N^{\delta1}$, $^{1}H^{\delta2}$-$^{15}N^{\varepsilon2}$, and $^{1}H^{\delta2}$-$^{15}N^{\delta1}$ signals are appeared, indicating the imidazole ring of His205 is in charged state. Similarly, His53 was also found in charged state. For His102 and His170, the chemical shift differences between $^{15}N^{\delta1}$ and $^{15}N^{\varepsilon2}$ are much larger, and the $^{1}H^{\delta2}$-$^{15}N^{\delta1}$ signal is missing, consistent with the $N^{\varepsilon2}$-H tautomer. Interestingly, His115 showed broad multiple peaks, which indicates that there are multiple conformations in slow-to-medium timescale exchange for its imidazole ring. With increased temperatures, the ^{1}H-^{15}N signals of His115 became less broadened, suggesting that multi-conformational exchange rate is getting faster. The chemical shifts and correlation pattern of His115 sidechain at 308K shows that the inmidazole should mainly exist as a $N^{\varepsilon2}$-H neutral tautomer in solution.

NMR spectroscopy for structure determination

The NMR sample of C-CD147^{EC} contained 0.8 mM ^{15}N, ^{13}C-labled protein in 50 mM PBS (pH 7.0) with 90% H_2O / 10% D_2O, along with 50 mM NaCl, 5 mM EDTA, 0.01% DSS, 0.01% NaN_3. All NMR experiments were collected on Bruker Avance 500, 600 and 800 MHz NMR (Bruker, Germany) spectrometers with cryoprobes at

298 K.

The backbone resonance assignments were obtained based on 2D ^{1}H-^{15}N HSQC, 3D HNCO, HNCACB, CBCA(CO)NH experiments. The aliphatic side-chain resonance assignments were obtained based on 2D ^{1}H-^{13}C HSQC, 3D (H) CCH-COSY, (H)CCH-TOCSY, HCCH-COSY and HCCH-TOCSY spectra. 3D ^{1}H-^{13}C NOESY-HSQC spectra and ^{1}H-^{15}N NOESY-HSQC spectra were acquired with mixing time of 120 ms, and then used for assignments confirmation and structure calculation. All NMR spectra were processed using NMRPipe (Delaglio et al., 1995) and analyzed with NMRView (Johnson and Blevins, 1994).

Structure calculation

The distance restraints were obtained by analyzing the NOSEY spectra and dihedral angle restraints were obtained by TALOS (Cornilescu et al., 1999). Initial structures were generated by CANDID (Herrmann et al., 2002) which were used as filter models for automated NOE assignments with SANE (Duggan et al., 2001), then we obtained refined NOE assignments and distance restraints. Those refined restraints were then used in DYANA module of CYANA 2.1 (Guntert et al., 1997) with standard CYANA simulated annealing schedule to gain refined structures. Then 100 structures with the lowest target function values among the 200 calculated structures were selected for the further AMBER12 refinement. Finally, SANE-AMBER calculation was carried out until no angle violation was bigger than 5° and no distance violation bigger than 0.2 Å. Twenty structures with the lowest AMBER energies were selected and a mean structure was generated by SUPPOSE. PROCHECK_NMR (Laskowski et

al., 1996) and MOLMOL (or PyMOL) were used to analyze the structure (Koradi et al., 1996).

NMR titration experiments of glycans

CD147^{EC} and three polysaccharides (N, N'-Diacetylchitobiose, 3'-sialyllactose sodium salt, 3α, 6α-mannopentaose) and five monosaccharides (sialic acid, D-mannose， D-glucose, Glucosamine hydrochloride, D-galactose) were used in NMR titration experiments. The NMR sample used for glycan titration contained 0.1 mM ^{15}N-labled CD147^{EC} in 20 mM MOPS (PH 7.0) with 90% H_2O / 10% D_2O, along with 50 mM NaCl, 0.01% DSS, 0.01% NaN_3. A series of 2D ^{1}H-^{15}N HSQC spectra with the increasing glycan concentrations were carried out at 298K on a Bruker Avance 700 MHz spectrometer with cryoprobe. The intensity and the chemical shift of all residues were analyzed.

Relaxation experiments and Model-free analysis

For relaxation measurements of C-CD147^{EC} with or without Zn(II), the backbone ^{15}N relation parameters including steady-state heteronuclear {^{1}H}-^{15}N NOE, the longitudinal relaxation rates (R_1) and transverse relaxation rates (R_2) were collected at 298K on a Bruker Avance 700 MHz spectrometer with cryoprobe. All experiments were carried out using conventional HSQC scheme as previous described (Farrow et al., 1994), but there was some difference in solvent suppression. Our programs used water flip-back for solvent suppression (Chen and Tjandra, 2011). The {^{1}H}-^{15}N NOE experiments were performed in the presence and absence of a 3 s proton presaturation period prior to the ^{15}N excitation pulse and using recycle delays of 2 and 5 s,

respectively (Renner et al., 2002). For C-CD147^{EC} without Zn(II), the delays used for R_1 experiments were 10, 100 (×2), 200, 300, 400, 600, 800, 1000, 1200, 1500, 1800, 2100 and 2500 ms. The delays used for the R_2 experiments were 8 (×2), 16, 24, 32, 40, 48, 64, 80, 100, 125, 150, 200 and 250 ms. For C-CD147^{EC} with Zn(II), the delays used for R_1 experiments were 10, 100 (×2), 200, 300, 400, 500, 700, 900, 1200, 1600 and 2000 ms. The delay used for the R_2 experiments were 9 (×2), 18, 27, 36, 45, 54, 63, 72, 90, 117 and 153 ms. The relaxation rate constants (R_1 and R_2) were obtained by fitting the peak intensities to a single exponential function using the nonlinear least-squares method (Fushman et al., 1997). The internal dynamic parameters were analyzed using the Model-free formalism.

Table S1. Restraints and structural statistics of C-CD147^{EC}.

NOE restraints	5088
Intraresidue	1237
Sequential	747
Medium-range	300
Long-range	1247
Ambiguous	1557
Dihedral angle restraints	146
φ angle	76
ψ angle	70
Chirality restraints	378
ω angle	106
Side chain	272
Structural Statistics	
Violations	
Distance restrains (> 0.2 Å)	1
Dihedral angle restraints	0
RMSD from mean	
Backbone heavy atoms(secondary structure region)	0.187 ± 0.037
All heavy atoms (secondary structure region)	0.739 ± 0.064
PROCHECK	
Most favored regions (%)	87.9
Additionally allowed regions	12.0
Generously allowed regions	0.1
Disallowed regions	0.1

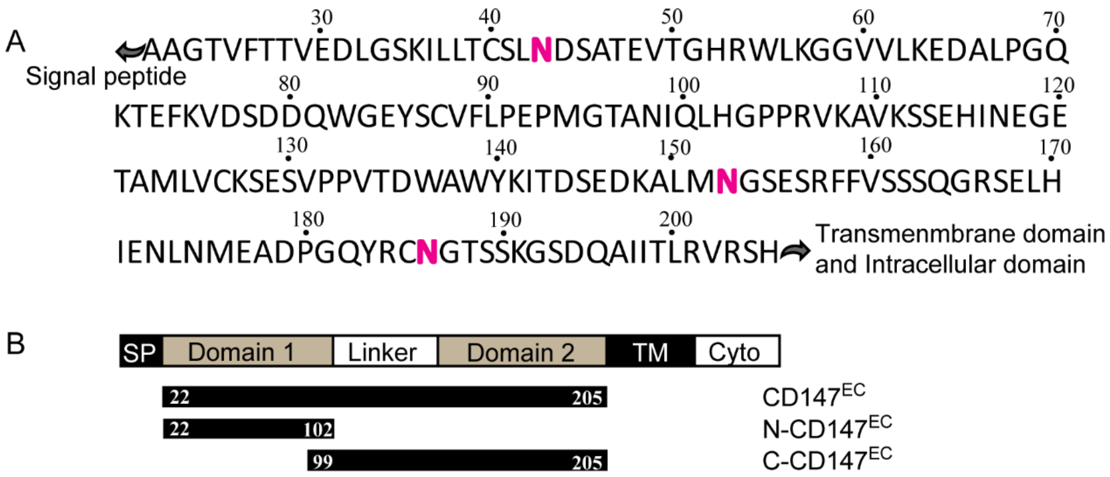

Figure S1. Recombinant CD147^{EC} expressed in *E.coli*. (A) Amino acids sequence of the extracellular portion of CD147 (CD147^{EC}). The glycosylation sites are labeled in Magenta. (B) The schematic domain organization of CD147 and the CD147^{EC} constructs used in this study.

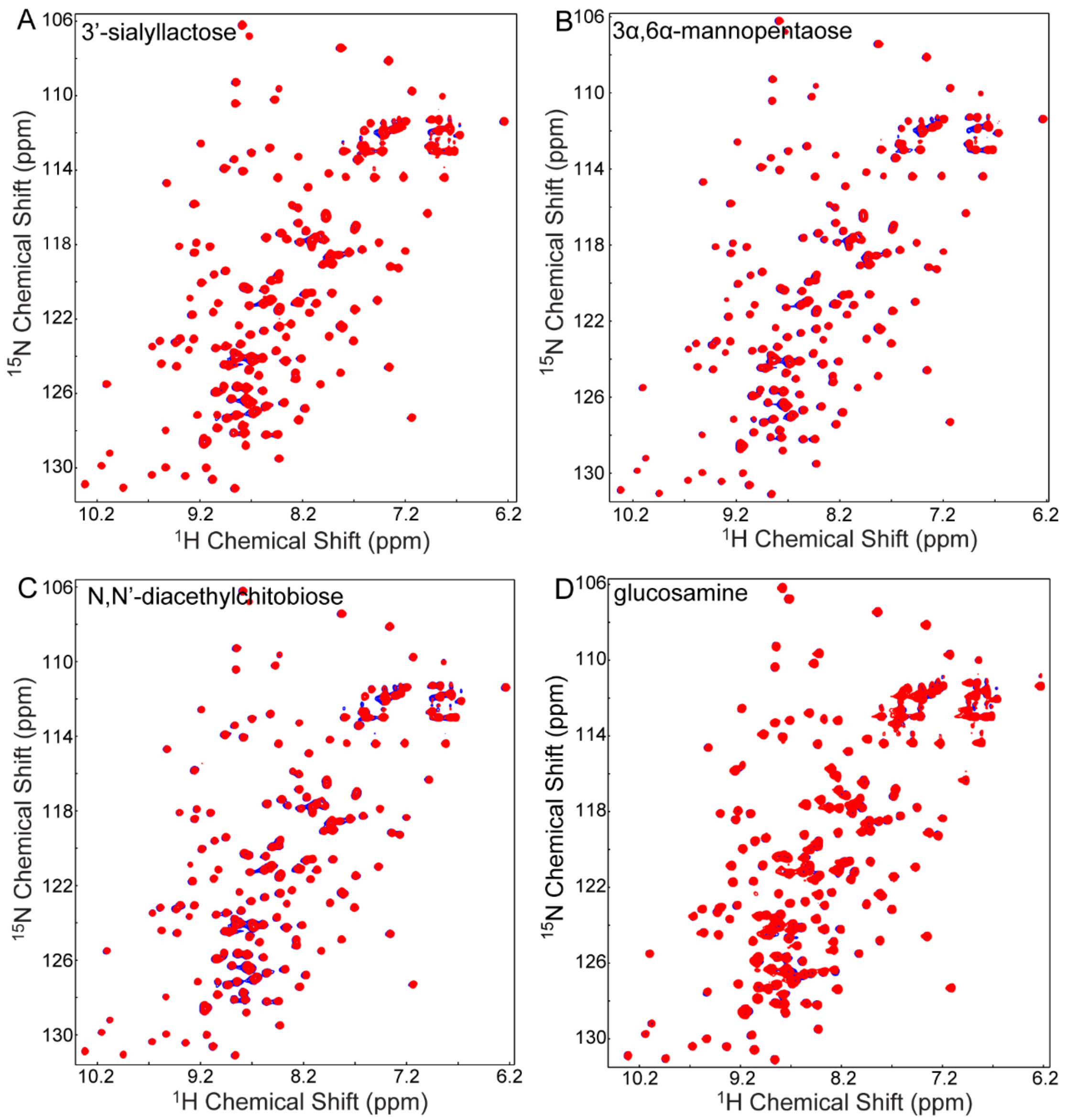

Figure S2. CD147^{EC} does not interact with glycans. Overlay of 2D ^{1}H-^{15}N HSQC spectra of CD147^{EC} in the absence (blue) and in the presence (red) of glycans. (A) 3'-siallyllactose. (B) 3α,6α-mannopentaose. (C) N, N'-diacethylchitobiose. (D) Glucosamine.

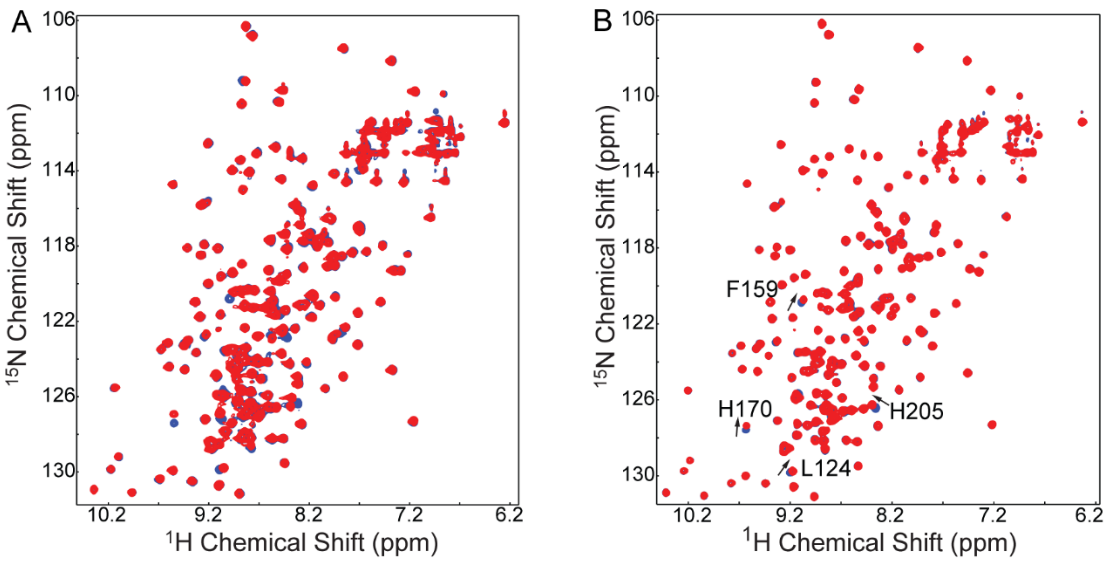

Figure S3. (A) 2D ^{1}H-^{15}N HSQC spectra under different pH conditions, blue (pH 6.5), red (pH 7.0). (B) Overlay of 2D ^{1}H-^{15}N HSQC spectra of CD147^{EC} in the absence (blue) and in the presence (red) of 10-fold of sialic acid. Residues showing significant chemical shift changes are indicated.

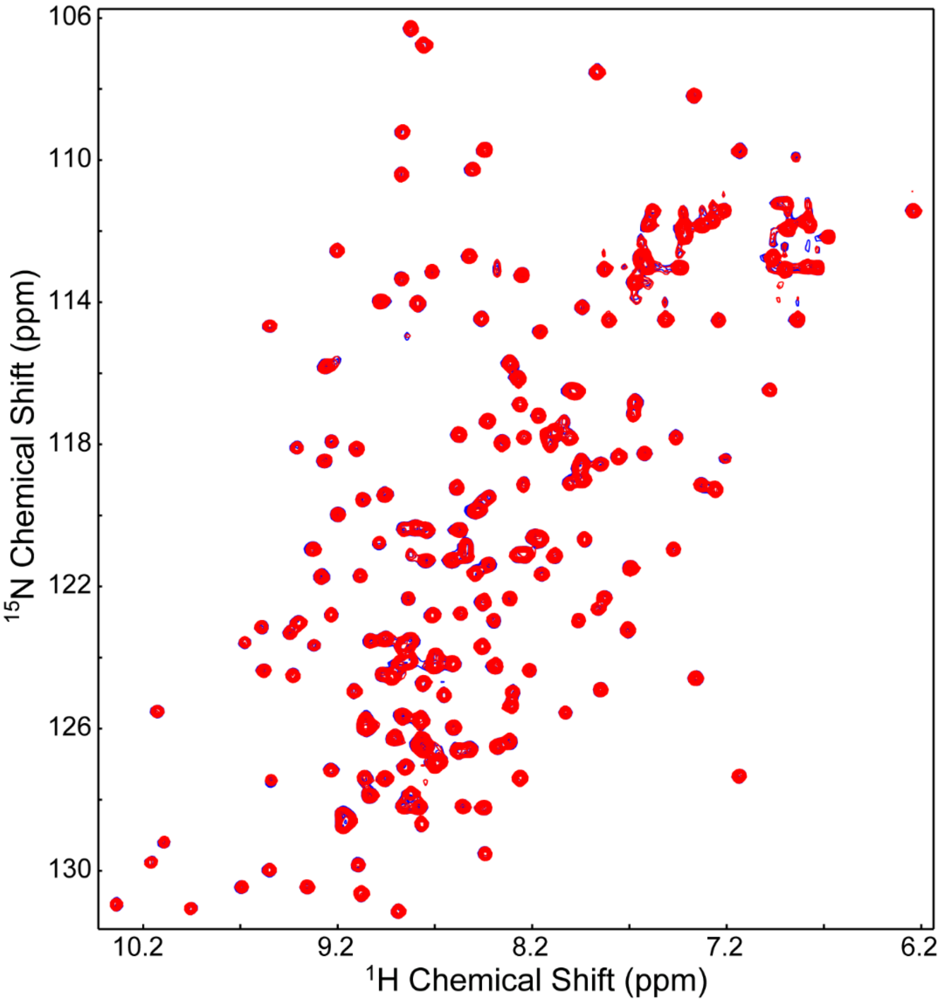

Figure S4. NH signal intensity reduction caused by Zn^{2+} can be rescued by EDTA.

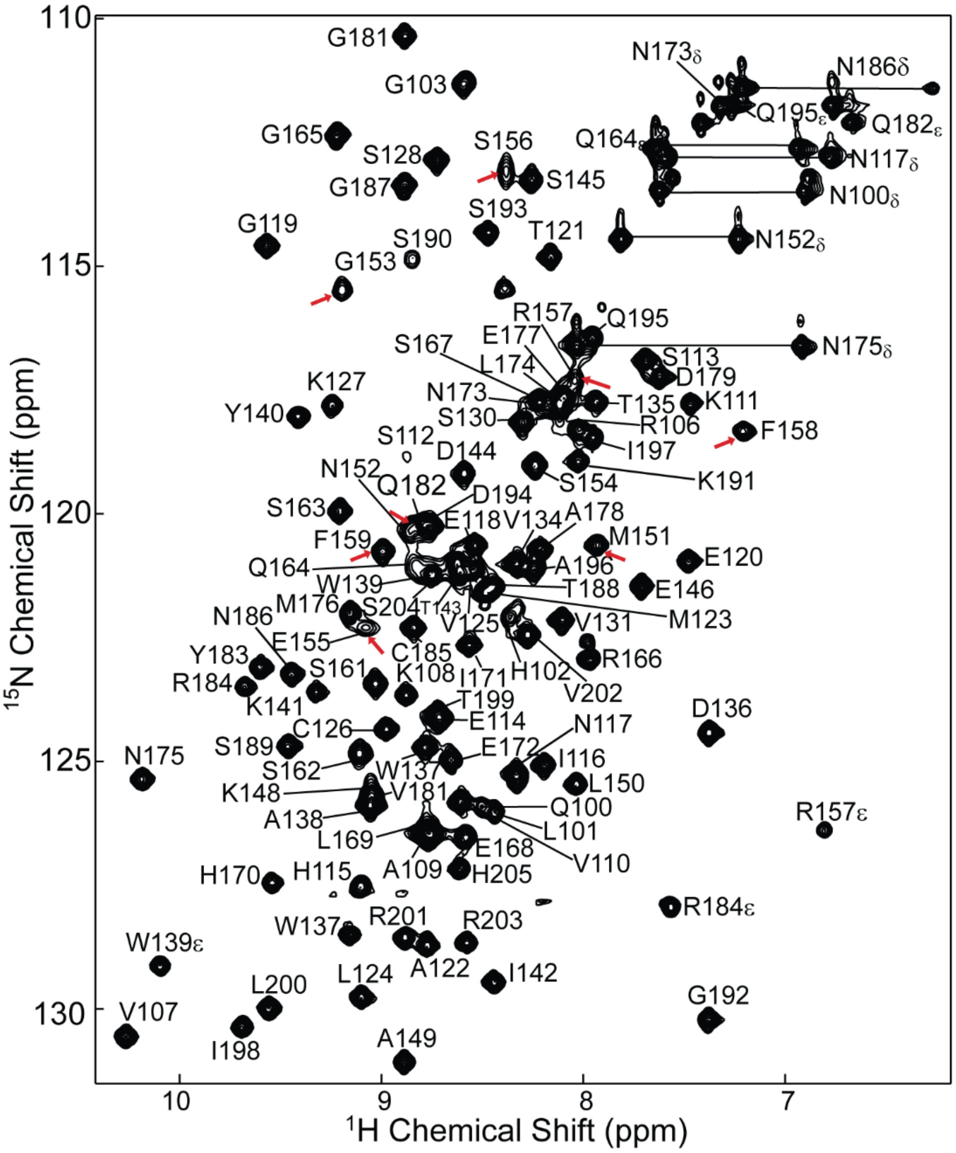

Figure S5. 2D ^{1}H-^{15}N HSQC spectrum of C-CD147^{EC} with resonance assignments indicated.

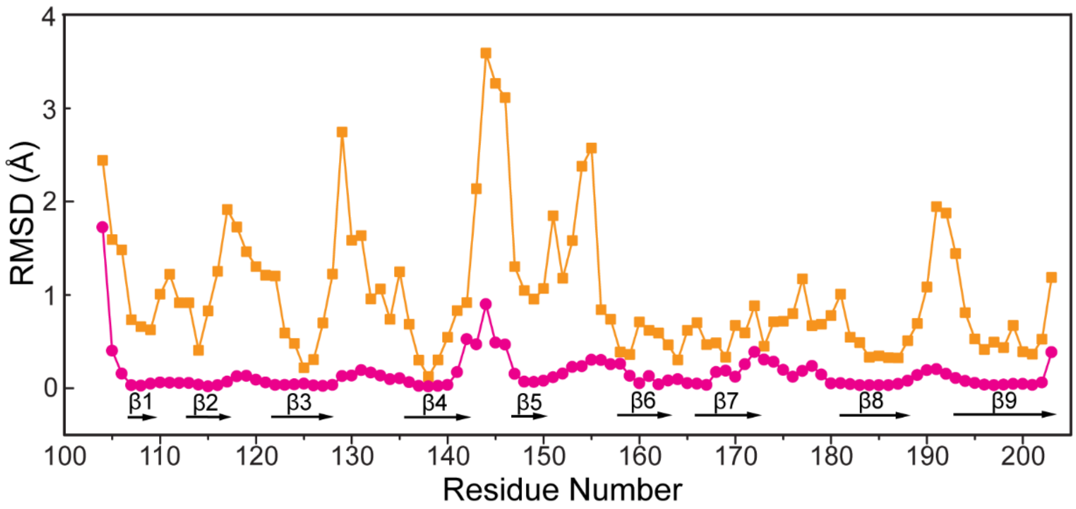

Figure S6. Per-residue RMSD of the solution structure ensemble (magenta) verses per-residue RMSD between the mean solution structure and the crystal structure (yellow, PDB ID: 3B5H).

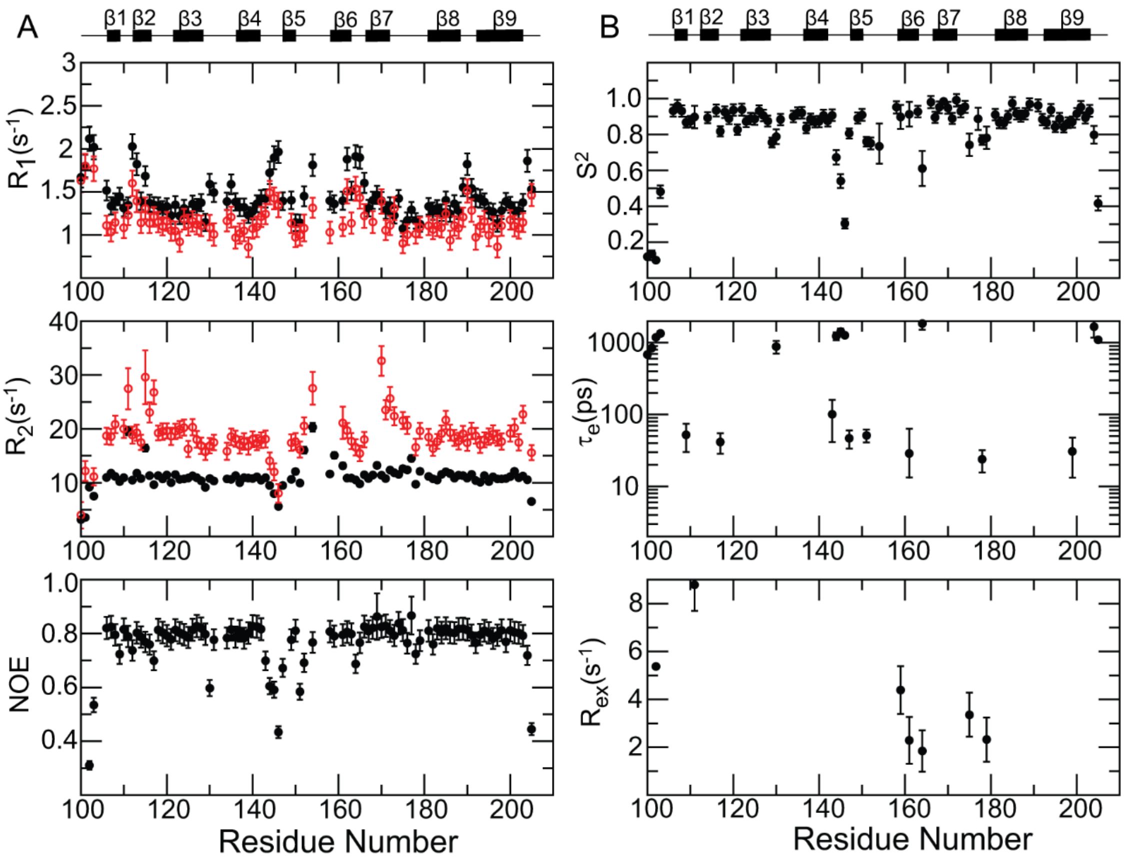

Figure S7. Backbone relaxation data and internal mobility parameters of C-CD147^{EC}. (A) Longitudinal relaxation rates (R_1), transverse relaxation rates (R_2) and heteronuclear {^{1}H}-^{15}N NOE values of the C-CD147^{EC}. R_1 and R_2 of C-CD147^{EC} with Zn(II) are colored in red and those without Zn(II) are colored in black. (B) Internal mobility parameters of generalized order parameter S^2, internal correlation time τ_e, the conformational exchange R_{ex} of the C-CD147^{EC} without Zn(II). The spectra for determining the relaxation parameters were recorded on a Bruker Avance 700 MHz spectrometer at 25 °C. The samples (0.8 mM) were dissolved in 50 mM phosphate buffer containing 50 mM NaCl and 5 mM EDTA at pH 7.0. Secondary-structure elements are shown on top.

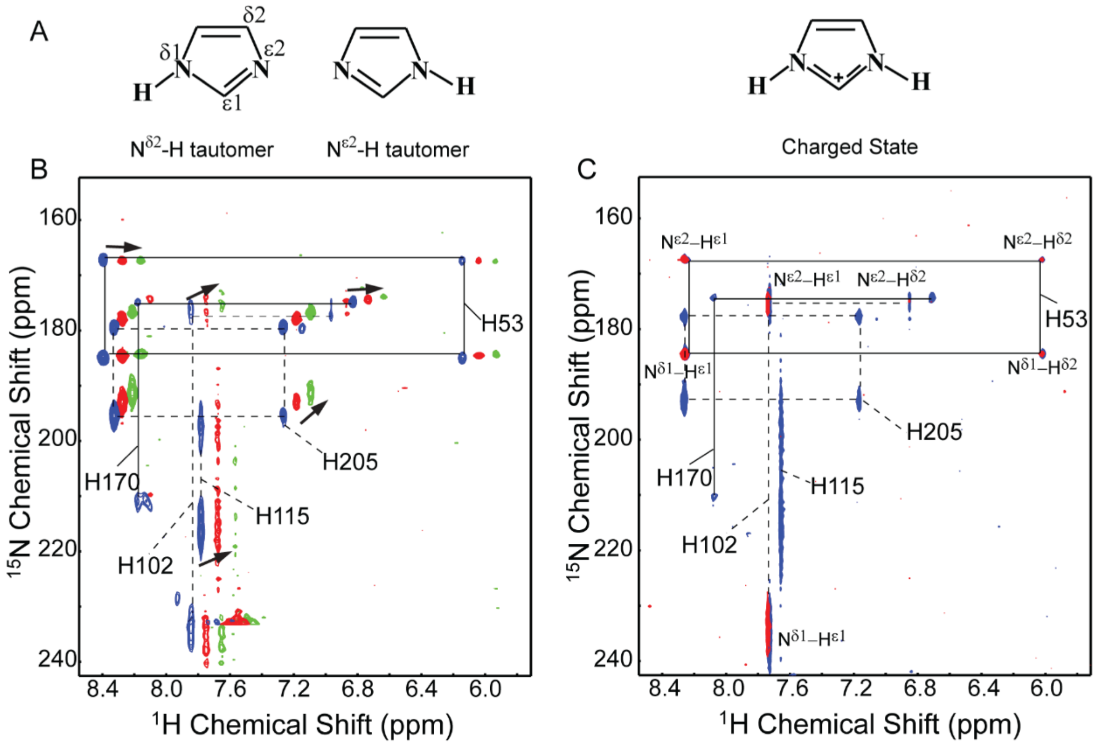

D

	$N^{\delta1}$(ppm)	$H^{\varepsilon1}$(ppm)	$N^{\varepsilon2}$(ppm)	$H^{\delta2}$(ppm)
His53	184.2	8.26	167.4	6.02
His102	234.5	7.73	175.6	6.71
His115	188-203	7.78	209-224	NA
His170	210.5	8.07	174.5	6.71
His205	193.1	8.26	177.6	7.16

Figure S8. Analysis of tautomeric states of histidine side-chains. (A) The nomenclature used to describe the imidazole atoms of histidine. Three possible protonation states of the histidine ring. Form I and form II are neutral tautomeric states, and another state is charged form. (B) 2D ^{1}H-^{15}N HSQC spectra showing multi-bond ^{1}H and ^{15}N correlation signals of histidine sidechains at different temperature, 288K(blue), 298K(red) and 308K (green). The direction of arrow represents temperature decrease. (C) 2D ^{1}H-^{15}N HSQC spectra showing multi-bond ^{1}H and ^{15}N correlation signals of histidine sidechains of CD147^{EC} with (red) or without (blue) Zn(II) at 298K. The cross-peak patterns for His53 (solid line), His102 (dashed line), His115 (dashed line), His170 (solid line) and His205 (dashed line) are

shown. (D) Histidine imidazole ^{15}N and ^{1}H chemical shifts of $CD147^{EC}$ at 298K. NA, not available.
